# Less is More: Design of a Highly Stable Disulfide-Deleted Mutant of Analgesic Cyclic α-Conotoxin Vc1.1

**DOI:** 10.1038/srep13264

**Published:** 2015-08-20

**Authors:** Rilei Yu, Victoria A. L. Seymour, Géza Berecki, Xinying Jia, Muharrem Akcan, David J. Adams, Quentin Kaas, David J. Craik

**Affiliations:** 1 Institute for Molecular Bioscience, The University of Queensland, Brisbane, Queensland, 4072, Australia; 2 Health Innovations Research Institute, RMIT University, Melbourne, Victoria, 3083, Australia

## Abstract

Cyclic α-conotoxin Vc1.1 (cVc1.1) is an orally active peptide with analgesic activity in rat models of neuropathic pain. It has two disulfide bonds, which can have three different connectivities, one of which is the native and active form. In this study we used computational modeling and nuclear magnetic resonance to design a disulfide-deleted mutant of cVc1.1, [C2H,C8F]cVc1.1, which has a larger hydrophobic core than cVc1.1 and, potentially, additional surface salt bridge interactions. The new variant, hcVc1.1, has similar structure and serum stability to cVc1.1 and is highly stable at a wide range of pH and temperatures. Remarkably, hcVc1.1 also has similar selectivity to cVc1.1, as it inhibited recombinant human α9α10 nicotinic acetylcholine receptor-mediated currents with an IC_50_ of 13 μM and rat N-type (Ca_v_2.2) and recombinant human Ca_v_2.3 calcium channels via GABA_B_ receptor activation, with an IC_50_ of ~900 pM. Compared to cVc1.1, the potency of hcVc1.1 is reduced three-fold at both analgesic targets, whereas previous attempts to replace Vc1.1 disulfide bonds by non-reducible dicarba linkages resulted in at least 30-fold decreased activity. Because it has only one disulfide bond, hcVc1.1 is not subject to disulfide bond shuffling and does not form multiple isomers during peptide synthesis.

Conotoxins are disulfide rich peptide toxins produced by marine cone snail belonging to the *Conus* genus^1^,^2^,^3^,^4^,^5^. α-Conotoxins are a subgroup of conotoxins characterized by their ability to inhibit nicotinic acetylcholine receptors (nAChRs)^4^,^5^,^6^. One α-conotoxin identified in the venom of *Conus victoriae*, Vc1.1^7^, has attracted considerable interest for its potent analgesic activity in two models of peripheral neuropathy of the rat sciatic nerve^8^, and it is currently being developed as a drug for treating neuropathic pain^9^. Vc1.1 is a 16-residue peptide, and its three-dimensional structure comprises a small α-helix as well as four cysteines forming two disulfide bridges and defining two loops^10^. The molecular target responsible for the analgesic activity of Vc1.1 has not yet been clearly identified^11^,^12^, as it potently inhibits both the rat α9α10 nAChR^10^,^13^ and N-type (Ca_v_2.2) and R-type (Ca_v_2.3) calcium channel currents via the GABA_B_ pathway^12^,^14^.

Recently, an orally active cyclic Vc1.1 (cVc1.1, Fig. 1) was engineered by joining the N- and C-termini of the peptide without affecting the three-dimensional structure or biological activity^9^. This molecule was designed because a major obstacle generally impeding the use of bioactive peptides as drugs is their high susceptibility to enzymatic degradation^15^,^16^. One strategy to improve the stability of peptides is to cyclize their N- and C-termini^16^,^17^,^18^,^19^, and this strategy has been used successfully with several classes of conotoxins^9^,^19^,^20^. cVc1.1 is the first orally active α-conotoxin variant and its potent activity makes it an attractive candidate for the development of new analgesics^9^.

The four cysteines in the peptide primary sequence of cVc1.1 can theoretically form three disulfide-bond isomers, with one of them being active. In general, the formation of multiple isomers complicates synthesis procedures and significantly increases the cost of production of peptides. It has been shown for other disulfide-rich conotoxins that only selected disulfide bonds are crucial for stability and activity^21^,^22^,^23^,^24^,^25^. Thus, removing one disulfide-bond of cVc1.1 might not affect its conformation and activity, especially if the cystine is substituted by judiciously chosen amino acids. To test this hypothesis, we used *in silico* modeling to design disulfide deleted variants and electrophysiology recording to study the activity of the resulting lead peptide. The new Vc1.1 analogue, [C2H,C8F]cVc1.1 has similar three-dimensional structure and activity to Vc1.1. However, since it has only one possible disulfide isomer, the cost of peptide synthesis and purification is reduced compared to the parent peptide. Specifically, crude cVc1.1 folds into two isomers in a 72:28 ratio^9^, whereas [C2H,C8F]cVc1.1 forms only one isomer, gaining an immediate improvement of 28% in folding yield.

## Results

### Design of cVc1.1 variants

**Figure 1:**
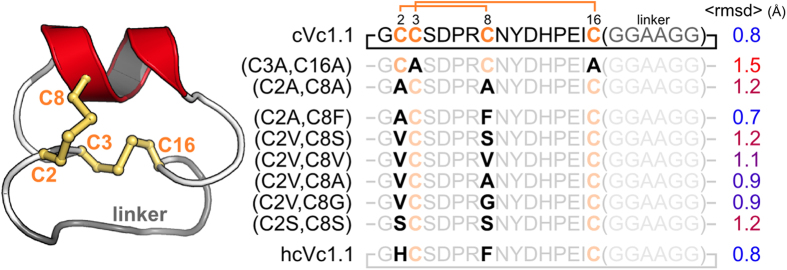
Solution structure of cVc1.1 and sequences of cVc1.1 wild-type and variants considered in this study. cVc1.1 is an engineered peptide in which a cyclizing linker (grey) was added to confer oral activity to the analgesic peptide Vc1.1. This peptide comprises two disulfide bonds, which are shown in orange. The substituted positions are shown in bold. The time-averaged backbone root-mean-square deviations (<rmsd>) from cVc1.1 NMR solution structure during 30 ns molecular dynamics simulations are indicated on the right. The conserved positions of the cVc1.1 variants are shown using lighter color fonts to highlight the substituted positions.

In the first step of the design process, molecular dynamics was used to determine which disulfide bond might be removed without affecting the stability of cVc1.1 (Fig. 1 and S1). Molecular dynamics simulations over 30 ns were performed for the two variants that have a pair of hemi-cystine residues replaced by alanines. The conformation of [C3A,C16A]cVc1.1 deviated from the NMR solution structure of cVc1.1 over the course of the simulation, with the Cα root-mean-square deviation (RMSD) between core regions of the mutant peptide and cVc1.1 on average 1.5 Å (range 1.0–2.0 Å). By contrast, the structure of [C2A,C8A]cVc1.1 was more similar to that of cVc1.1, with the Cα RMSD being only 1.2 Å (range 0.5–1.5 Å) (Fig. 1). Therefore, the disulfide bond between positions 3 and 16 seems more important for the stability of cVc1.1 than the disulfide bond between positions 2 and 8.

In a second round of *in silico* design, various types of residues were introduced at positions 2 and 8 to minimize the effect of the disulfide bond deletion on the global conformation of cVc1.1 (Fig. 1). The simulations suggested that introducing a Phe residue at position 8 and either a His residue or an Ala residue at position 2 stabilizes the core region of the peptide. The Cα RMSDs of these variants were of 0.8 and 0.7 Å, respectively, which is comparable to the change in Cα RMSDs observed during similar simulations of cVc1.1 (Fig. 1). The aromatic residue Phe introduced at position 8 stabilized the α-helix during the simulations by forming a hydrophobic cluster with residues Cys-3, His-12, Ile-15, and Cys-16. The final model suggested that a positively charged His residue at position 2 can potentially form a cation-π interaction with Phe-8 and a charge interaction with Asp-5. Overall, the computational data suggested that [C2H,C8F]cVc1.1 is as stable as cVc1.1. Since the new peptide contains a more hydrophobic core relative to the parent peptide we coined it hcVc1.1.

### NMR solution structure of hcVc1.1

**Figure 2:**
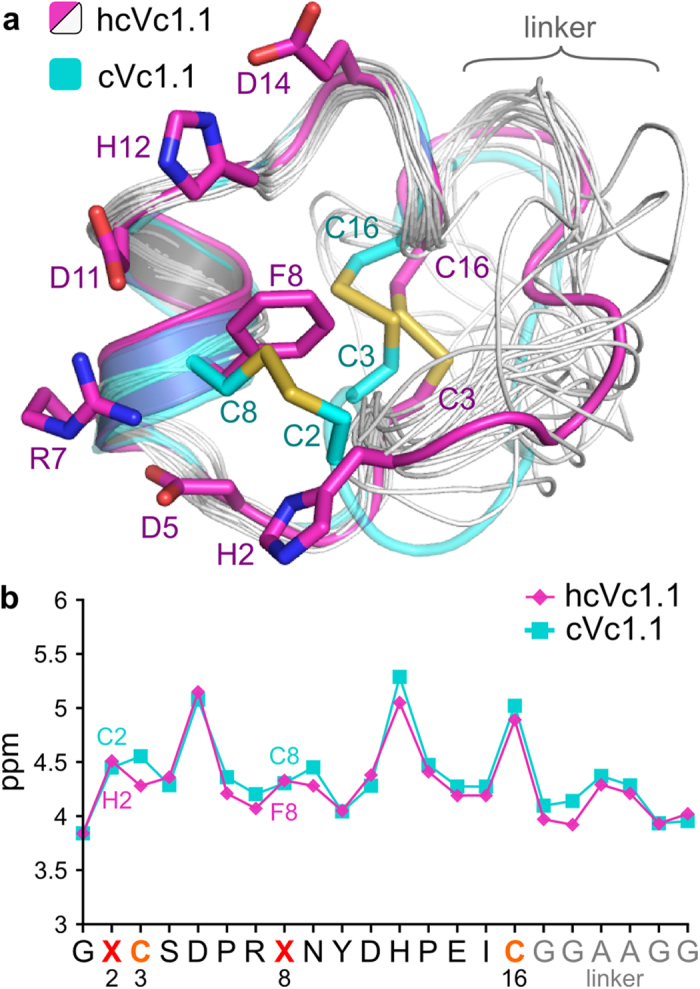
Comparison of the NMR solution structures of hcVc1.1 (pink and gray) and cVc1.1 (blue). (**a**) superimposition of the 20 minimum energy NMR models of hcVc1.1 and of the first NMR model of cVc1.1; the first lowest energy model of hcVc1.1 is in pink and the trace of the other models are in gray; the cVc1.1 lowest energy model is in blue. (**b**) Hα chemical shifts of hcVc1.1 and cVc1.1.

The three-dimensional solution structure of hcVc1.1 was determined using 22 dihedral angles and 135 distance restraints, including 54 sequential, 56 medium and 25 long range NOEs. The backbone amide hydrogens of residues Asp-5, Phe-8, Tyr-10, Asp-11, His-12 and Ile-15 appear to be involved in hydrogen bond interactions, as judged by a hydrogen-deuterium exchange experiment monitored by NMR spectroscopy. The 20 lowest energy models of hcVc1.1 are shown in Fig. 2a. The backbone conformation of the peptide segment 3–16, which corresponds to Vc1.1, is well-defined, with a maximum Cα RMSD of 0.3 Å between NMR models, whereas the linker region of the peptide is more flexible.

A comparison of Hα chemical shifts of hcVc1.1 and cVc1.1, shown in Fig. 2b, indicates that the two peptides adopt very similar globular conformations. The chemical shift deviation of 0.3 ppm observed at position 3 probably originates from the shielding effect of the aromatic ring current of Phe-8^26^. The Hα of Cys-3 is indeed ideally located to be shielded as it is in the same plane as the Phe-8 side chain and is 4.5 Å from the center of the phenyl ring (Fig. 2). The iso-shielding lines described by Johnson and Bovey predict a shift of 0.3 ppm downfield of this proton^26^, in excellent agreement with the measured difference between cVc1.1 and hcVc1.1. The backbone conformation of the hcVc1.1 NMR model is similar to that of cVc1.1 (Fig. 2a), with the core region of the peptide superimposing with a backbone RMSD of 0.8 Å.

**Figure 3:**
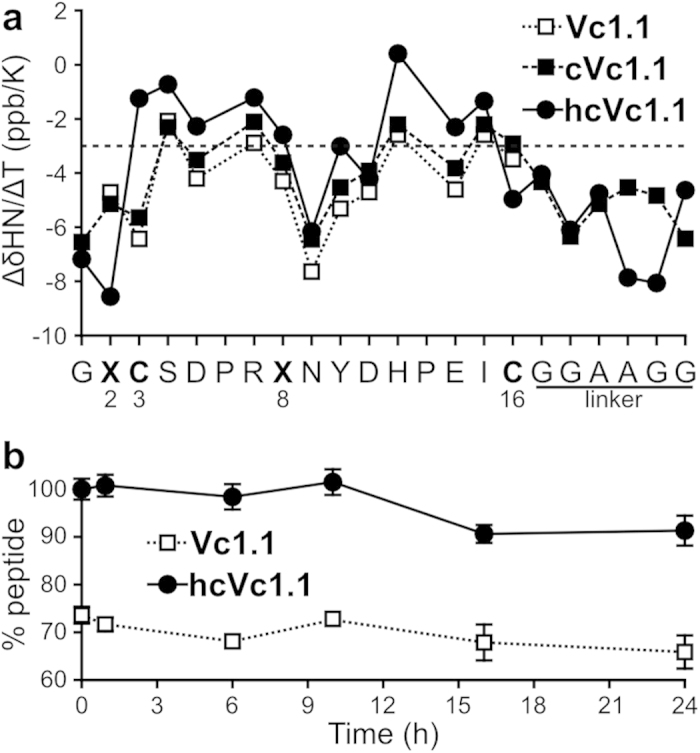
(**a**) Comparison of the amide temperature coefficients of the backbone amid hydrogens of Vc1.1, cVc1.1 and hcVc1.1 (values are in Table S3). (**b**) Serum stability of Vc1.1 and hVc1.1 measured as percentage of peptide remaining in serum. The drop of Vc1.1 remaining at t = 0 is due to disulfide shuffling to an alternative disulfide isomer^9^.

The protection of amide protons from solvent can be assessed with amide temperature coefficients (ΔδHN/ΔT), and this in turn provides information about the internal hydrogen bond network^27^. A comparison of ΔδHN/ΔT values between hcVc1,1, Vc1.1 and cVc1.1, shown in Fig. 3a, suggests that the internal hydrogen bond network of hcVc1.1 is slightly better defined than those of the two other peptides. Indeed, the ΔδHN/ΔT values for residues between positions 3 and 15 (except for positions 9 and 11) of hcVc1.1 are above -3 ppm/K and are more positive than those of Vc1.1 and cVc1.1^9^,^10^. Notably, the hydrogen bonds established by the backbone of modified position 8 are also more stable.

### Chaotropic and enzymatic stability of hcVc1.1

The stability of hcVc1.1 over a range of temperatures and pH values was monitored by NMR spectroscopy. Spectra used for solution structure determination of hcVc1.1 were recorded at 280 K at pH 4.5. The Hα chemical shift remained the same for all temperatures from 280 K to 310 K and for all pH values from 3.1 to 6.0 (Fig. S3). At pH values above 6.5, the peaks of some residues start to disappear because of fast amide proton exchange with the solvent. The insensitivity of the Hα chemical shifts to pH and temperature changes suggests that the peptide fold is maintained at physiological temperature and pH conditions.

In human serum, hcVc1.1 was significantly more stable than Vc1.1 (Fig. 3b). After 24 h, about 90% of the initial peptide remains for hcVc1.1, which is similar to the stability of cVc1.1^9^. Therefore, hcVc1.1 is stable and resistant to enzymatic degradation at physiological conditions, *i.e*. at pH 7.0 and 310 K.

### hcVc1.1 inhibition of recombinant human α9α10 nAChRs in Xenopus oocytes

**Figure 4:**
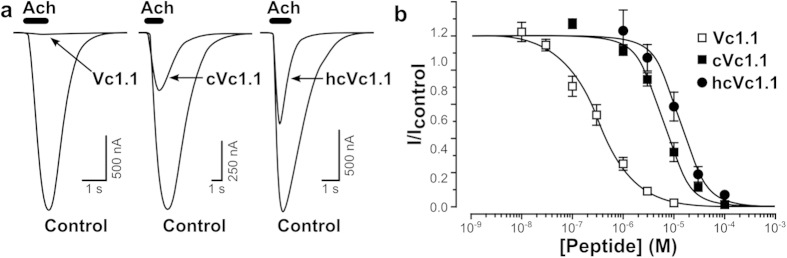
Activity of hcVc1.1 at the α9α10 nAChR. (**a,b**). (**a**) Superimposed representative traces of ACh (10 μM)-evoked inward currents obtained in the absence (control) and presence of 1 μM Vc1.1, cVc1.1 and hcVc1.1 applied to hα9α10 nAChRs expressed in oocytes. (**b**) Concentration-response curves for inhibition of hα9α10 currents by Vc1.1, cVc1.1 and hcVc1.1. Data points represent relative peak current amplitudes (I/Icontrol), mean ± SEM; n = 3–7. IC_50_ values obtained for Vc1.1, cVc1.1 and hcVc1.1 are 320 nM, 6 μM and 13 μM, respectively.

**Figure 5:**
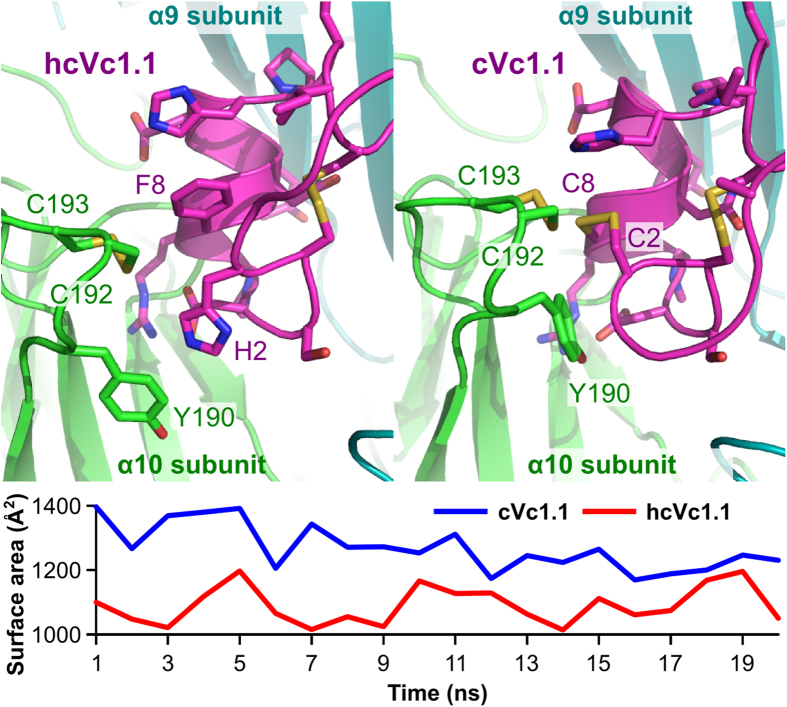
Conformations of the interactions between hcVc1.1 (top left) or cVc1.1 (top right) and hα9α10 nAChR during after 20 ns molecular dynamic simulations. The evolution of the buried surface area (Surface area) is shown in the bottom graph over the simulation.

We previously reported that Vc1.1 and cVc1.1 inhibit rat α9α10 nAChRs in a concentration-dependent manner with IC_50_ values of 64 nM and 765 nM, respectively^9^,^12^. In the present study, Vc1.1, cVc1.1 and hcVc1.1 were examined at human α9α10 nAChRs expressed in *Xenopus* oocytes. The differential effect of 1 μM Vc1.1, cVc1.1 or hcVc1.1 on inhibition of the ACh (10 μM)-evoked current amplitude is shown in Fig 4a. ACh-evoked current amplitude was inhibited in a concentration-dependent manner by Vc1.1, cVc1.1 or hcVc1.1 with the corresponding concentration-response curves giving IC_50_ values of 320 nM, 6 μM and 13 μM (n = 3–7), respectively (Fig. 4b). The potency of Vc1.1 and cVc1.1 at human α9α10 nAChRs was reduced 5- to 8-fold compared to inhibition of rat α9α10 nAChRs, potentially reflecting differences in the α9 extracellular domain^28^. α-Conotoxin RgIA is 300-fold less potent on the human versus rat α9α10 receptor, and mutational studies indicated that the primary determinant of this disparity is a single amino acid difference between the rat and human α9 nAChR subunit at position 59^29^. In a previous study^30^, we suggested that position 59 was also key to the difference in activity of Vc1.1 at inhibiting rat and human α9α10 nAChR, and we suggested that the C-terminal amide of Vc1.1 makes a hydrogen bond with the side chain of rat α9 Thr-59 but not with human α9 Ile-59. A molecular dynamics simulation of the interactions between hcVc1.1 or cVc1.1 and human α9α10 (Fig. 5) carried out in the present study suggests that the higher activity of cVc1.1 compared to hcVc1.1 originates from a better complementarity at the interface, with an interface area of 1,264 ± 15 Å^2^ or 1,090 ± 13 Å^2^, respectively. By comparison, protease inhibitors have on average an interface area of 1,500 Å^2^^31^, and α-conotoxin ImI buries 1,600 Å^2^ surface area at the interface with α7 nAChR^32^. The lower interface area of cVc1.1 and hcVc1.1 seems therefore to correlate with their micromolar range activity.

### hcVc1.1 inhibition of human Ca_v_2.3 channels and rat N-type (Ca_v_2.2) channels via GABA_B_ receptor activation

**Figure 6:**
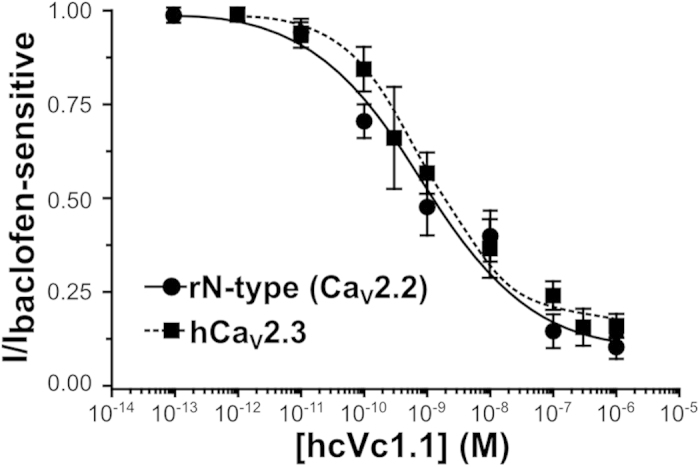
Concentration-response curves for inhibition by hcVc1.1 of rat N(rN)-type (Ca_v_2.2) channels in DRG neurons and recombinant human Ca_v_2.3 (hCa_v_2.3) channels co-expressed with human GABA_B_ receptors in HEK-293 cells. Barium ions at 2 mM and 10 mM were used as charge carrier (I_Ba_) for experiments with DRG neurons and hCav2.3, respectively. Baclofen (50 μM) was applied to determine the baclofen-sensitive I_Ba_ fraction. Data points representing mean ± SEM of peak I_Ba_ amplitude (n = 5–8 cells per data point) were plotted relative to the baclofen-sensitive I_Ba_ fraction (see Methods). The best fits with the Hill equation resulted in IC_50_ values of 857 ± 516 pM and 961 ± 254 pM for Ca_v_2.2 and hCa_v_2.3, respectively.

We recently demonstrated that cVc1.1 potently inhibits N-type (Ca_v_2.2) calcium channels in rat dorsal root ganglion (DRG) neurons and recombinant human Ca_v_2.3 channels expressed in human embryonic kidney (HEK-293) cells^9^,^33^. We have also shown that Vc1.1 inhibition in these cells is mediated via pertussis toxin-sensitive G protein-coupled GABA_B_ receptor signaling and is dependent on specific c-Src phosphorylation^14^,^33^. Here we show that hcVc1.1 also potently inhibits Ba^2+^ current through N-type (Ca_v_2.2) calcium channels in rat DRG neurons and recombinant human Ca_v_2.3 calcium channels co-expressed with human GABA_B_ receptors in HEK-293 cells (Fig. S4). We determined the hcVc1.1 concentration dependence of I_Ba_ inhibition for N-type (Ca_v_2.2) and Ca_v_2.3 channels (Fig. 6) and included the half-maximal inhibition concentration (IC_50_) values in Table 1.

These data demonstrate that hcVc1.1 inhibits human recombinant α9α10 nicotinic acetylcholine receptor (nAChR) currents with a two-fold lower potency than cVc1.1. In rat DRG neurons and HEK cells, hcVc1.1 had three-fold lower potency than cVc1.1, and inhibited Ba^2+^ currents through native N-type (Ca_v_2.2) calcium channels and recombinant human Ca_v_2.3 calcium channels, respectively (Table 1).

## Discussion

In this study we simplified the structure of cVc1.1 by removing one of its disulfide bonds while preserving its conformation, stability and selectivity. This new peptide was rationally designed in two steps: in the first step, a disulfide bond that could be deleted and yet cause minimal perturbation of the scaffold was identified. The largest loop of [C3A,C16A]cVc1.1 contains three more residues than the largest loop of [C2A,C8A]cVc1.1, and this size difference provides a simple explanation for the greater flexibility observed in molecular dynamics simulations of the cystine 3–16 substituted variant. In a second step, the nature of the amino acids used to substitute the cystine was optimized to increase stability. Our strategy consisted of extending the hydrophobic core, which is identified as an important stabilizing factor of mini-proteins^34^,^35^, and creating additional surface salt bridge interactions, which can in some cases stabilize proteins but in other cases can either have minimal or detrimental effects on stability^36^. The surface charged residues of hcVc1.1, *i.e*. His-2, Asp-5, Arg-7, Asp-11, His-12, and Glu-14, form a series of interconnected salt bridges. The theoretical pKa of His-2 side chain is predicted to be 6.5, consistent with a mainly charged side chain at neutral pH. In summary, the strategy to increase the stability of hcVc1.1 consisted of increasing the hydrophobic/hydrophilic differences between the core and surface positions. Additionally, the charged side chain of His-2 can also potentially establish a cation-π interaction with Phe-8, and this type of interaction was shown to have an energy of 2–5 kcal/mol^37^.

Remarkably, the disulfide-deleted hcVc1.1 has similar stability to the parent peptide at all tested temperatures and pH conditions as well as in human serum. This high stability is noteworthy because disulfide bonds are generally regarded as important for the stability of conotoxins^38^. A strategy consisting of creating a compact hydrophobic core was also employed to design the smallest peptide that can adopt a defined fold without disulfide bond, namely Trp-cage^35^. One possible advantage of stabilizing a peptide without using multiple disulfide bonds is to withstand harsher pH conditions and to easily refold upon mild denaturation. Shuffling of disulfides can indeed result in peptide degradation and significant loss of activity^38^. By contrast, hydrophobic cores are not easily disrupted by pH changes and peptides with small compact hydrophobic cores potentially have better stability *in vivo* than disulfide stabilized ones if they can resist enzymatic degradation.

The ability of hcVc1.1 to inhibit currents through human α9α10 nAChRs, rat N-type (Ca_v_2.2) and human Ca_v_2.3 channels is only slightly lower than that of cVc1.1. This result is in stark contrast with other attempts to modify the nature of Vc1.1 disulfide bonds by replacing them with dicarba bridges, resulting in ~30–100-fold decrease or loss of activity^39^. Interestingly, the solution structures of some dicarba analogues display nearly identical backbone conformations to cVc1.1, suggesting that the drop in activity is due to subtle modifications of the epitope presentation. Molecular modeling of the complex between hcVc1.1 and α9α10 nAChR suggests that hcVc1.1 and cVc1.1 have shape complementarity at the interface, which is remarkable because the 2–8 disulfide bond establishes extensive interactions at the interface. By contrast, the simulations of the interactions of Vc1.1 2–8 dicarba analogue with α9α10 nAChR suggested a possible loss of interaction^39^. The molecular details of the interaction between cVc1.1 and GABA_B_ is unknown, but the 3–16 disulfide bond rather than the 2–8 disulfide bond was proposed to be important for activity^39^, in agreement with hcVc1.1 displaying similar activity on the GABA_B_ pathway as cVc1.1.

The importance of the two disulfide bonds for activity differs between α-conotoxins. For example, the first loop^40^ and disulfide bond^41^ of α-conotoxin ImI (2–8) and not the second disulfide bond^42^ was shown to impact inhibition of nAChR α7^41^,^42^, in contrast with our results that show that the first disulfide bond of cVc1.1 could be modified without dramatically impacting its activity. Both ImI and cVc1.1 have four residues in their first loop, but their second loops have different lengths, with three and seven residues, respectively. This difference of loop length results in different peptide conformations and therefore interactions with nAChRs. ImI has a shorter α-helix than cVc1.1 and Vc1.1^9^,^10^,^43^, and this helix only establishes a limited number of interactions with the α7 nAChR^44^, resulting in the first loop and disulfide bond of ImI making most of the contacts with the receptor. By contrast, the α-helices of Vc1.1^30^, cVc1.1 and hcVc1.1 (this study) are more deeply buried at the interface than that of ImI (*e.g*. Tyr-10 of Vc1.1 is completely buried but Trp-10 of ImI is partly solvated)^44^, and this larger number and sequence distribution of interface residues for Vc1.1 and cVc1.1 probably make them more robust to modifications of the first disulfide bond and loop than ImI.

## Conclusion

Backbone cross-links, such as disulfide bonds or dicarba bridges, are widely used strategies to stabilize engineered peptides. We show here that rationally designed non-covalent interactions can stabilize the internal hydrogen bond network of a peptide scaffold. Remarkably, we were able to globally preserve the biological activity of a peptide despite swapping one of its disulfide bonds for residues that increase the hydrophilic/hydrophobic discrepancy between core and surface positions. Considering the structural simplicity and conformational stability of this new active peptide, hcVc1.1 is an improved lead molecule for the development of analgesic compounds for the treatment of neuropathic pain.

## Materials and Methods

### Computational modeling

The designs of cVc1.1 variants considered in this study are summarized in Fig. 1. Their structures were modeled by substituting the corresponding residues in cVc1.1 NMR structure^9^ using Modeller (version 9v7)^45^,^46^. The models and the first NMR structure were minimized and refined using molecular dynamics simulations (MD) performed with the Amber 10 package and the ff03 force field^47^,^48^. The peptides were solvated in a truncated octahedral TIP3P water box containing ~3,000 water molecules. Sodium ions were added to neutralize the systems. The systems were first minimized with 3,000 steps of steepest descent and then 3,000 steps of conjugate gradient with the solute restrained to their position by a harmonic force of 100 kcal/mol·Å^2^. A second minimization was then performed but with all position restraints withdrawn. The systems were then gradually heated up from 50 to 300 K in the NVT ensemble over 100 ps with the solute restrained to their position using a 5 kal/mol·Å^2^ harmonic force potential. The MD simulations were then carried out in the NPT ensemble, and the position restraints were gradually removed over 100 ps. The production runs were conducted over 30 ns simulation time with pressure coupling set at 1 atm and a constant temperature of 300 K. The MD simulations used a time step of 2 fs and, all bonds involving hydrogen atoms were maintained to their standard length using the SHAKE algorithm^49^. The particle-mesh Ewald (PME) method was used to model long-range electrostatic interactions^50^. Molecular models of the interactions of Vc1.1 and hcVc1.1 with human α9α10 nAChR ligand binding domains were prepared using an homology approach described previously^30^. The models were subjected to 20 ns unrestrained MD simulations using a similar protocol described above. MD trajectories were analyzed using VMD^51^ and molecules were drawn using PyMol (Schrödinger, LLC). The protonation states of side chains were evaluated using propka 3.1^52^.

### Peptide synthesis

hcVc1.1 was assembled manually by solid-phase peptide synthesis using Boc chemistry (Fig. S5). A Boc-Gly-PAM resin was used with the *in situ* neutralization/2-(1H-benzotriazol-1-yl)-1,1,3,3-tetramethyluronium hexafluorophosphate (HBTU) activation procedure^53^. Peptides were cleaved using hydrogen fluoride (HF), with *p*-cresol and *p*-thiocresol as scavengers [9:0.8:0.2 (vol/vol) HF/*p*-cresol/*p*-thiocresol] at 0 °C in an ice-water bath for 1.5 h. After cleavage, the peptides were precipitated with ice-cold ether, filtered, dissolved in 50% buffer A/B (buffer A: H_2_O/0.05% trifluoroacetic acid; buffer B: 90% CH3CN/10% H_2_O/0.045% trifluoroacetic acid), and lyophilized. Crude peptides were purified by reversed-phase HPLC (RP-HPLC) on a Phenomenex C18 column using a gradient of 0–75% buffer B in 75 min, with the eluent monitored at 214/280 nm. The same conditions were also used in the subsequent purification steps. Electrospray-mass spectroscopy was used to confirm the molecular mass of the linear peptide fractions before being pooled and lyophilized for oxidation. Cysteine residues were oxidized in one step in 0.1 M NH_4_HCO_3_ (pH 8 ~ 8.5) at a peptide concentration of 0.3 mg/ml with stirring overnight at room temperature. After oxidation, the peptides were purified by RP-HPLC using a gradient of 0–80% buffer B over 180 min. Analytical RP-HPLC and electrospray-mass spectroscopy confirmed the purity and molecular mass of the synthesized peptides (Fig. S6 and Table S1).

### NMR structure determination

hcVc1.1 NMR data were collected on a Bruker Avance 600 MHz spectrometer. The 2D experiments used for structure determination included TOCSY, NOESY, DQF-COSY and ECOSY in 90% H_2_O/10% D_2_O at 280 K, pH 4.5 with a mixing time of 300 ms. Peptide concentration was 1.7 mM and H chemical shifts were calibrated using DSS for all experiments. A D_2_O exchange experiment was performed to derive the backbone hydrogen bonds for structure calculation in 100% D_2_O at 280 K, pH 4.5. Hydrogen-deuterium exchange was monitored using 1D-1H NMR spectra recorded at 15 min, 5 h and 30 h. All NMR spectra were analyzed using CcpNmr^54^.

For structural model calculations, φ dihedral angles were derived from 2D DQF-COSY or 1D 1H NMR experiments using a strategy described by Clark *et al*.^9^. The φ angles were −60° ± 30 for His-2, Cys-3, Ser-4, Arg-7, Phe-8, Asn-9, Tyr-10, Asp-11, Glu-14, and Ile-15, and −120° ± 30 for Asp-5 and His-12. Additionally, the χ1 angles were 180° ± 30 for Cys-3, Asp-5, Phe-8, and Tyr-10, 60° ± 30 for Ser-4 and Asp-11, −60° ± 30 for His-12 and Cys-16, 60° ± 150 for Ile-15 and −60° ± 30 for His-2. The φ and χ1 dihedral angles were derived from the DQF-COSY and E-COSY experiments, respectively. Intra-residue NOE and ^3^J HN-Hα coupling patterns obtained from ECOSY spectra were used for the assignment of side chain dihedral angles. Hydrogen bond restraints were derived from D_2_O exchange experiments. Initial models of hcVc1.1 were computed using Cyana (version 3.0)^55^ to derive distance and dihedral restraints, which were used in a simulated annealing protocol implemented in CNS^56^ to generate 50 models in explicit water shells. The 20 structures with the lowest energies were selected as representatives of the solution structure of the peptide. A summary of the energy and geometry parameters of these models is shown in Table S2. The accuracy of the hcVc1.1 NMR models were evaluated using Molprobity^57^, as shown in Table S2.

### Temperature coefficients of hcVc1.1

hcVc1.1 was dissolved in 90% H_2_O/ 10% D_2_O at pH 4.5. The temperature was increased from 280 K to 310 K and the amide temperature coefficients were measured using 2D TOSCY experiments performed on a Bruker Avance 600 MHz spectrometer.

### Serum stability assay

Serum stability assay was performed using male AB human serum (Sigma-Aldrich), as described previously^9^. The serum was centrifuged at 15,000 g for 15 min to remove lipids; then it was incubated for 10 min at 37 °C. Triplicate samples were prepared at a 1:10 dilution of the peptide:serum with a working peptide concentration of 20 mM. 40 μL of 20% trifluoroacetic acid (TFA) was added to precipitate the serum proteins at 4 °C. Samples were centrifuged at 14,000 g for 10 min before analysis on a 0.3 mL/min Phenomenex C18 column using a linear 1% gradient of 0–50% solvent B. Triplicate samples of peptide in PBS were also run for each time point as controls. An aliquot of the sample was injected, and the amount of intact peptide remaining was determined by integration at 215 nm.

### cRNA preparation

Plasmid DNAs encoding human α9 and α10 subunits were linearized with appropriate restriction enzymes, and cRNA was synthesized *in vitro* using a T7 *in vitro* transcription kit (mMessage mMachine; Ambion, Foster City, CA).

### Oocyte preparation and microinjection

Stage V-VI oocytes were obtained from *Xenopus laevis*, defolliculated with 1.5 mg/ml collagenase (Type I, Sigma) in OR-2 solution (82.5 mM NaCl, 2 mM KCl, 1 mM MgCl_2_ and 5 mM HEPES at pH 7.4). Oocytes were injected with 5 ng cRNA for each of the human α9 and α10 subunits using an auto-nanoliter injector (Nanojet II, Drummond Scientific Co., Broomall, PA). Oocytes were incubated at 18 °C in sterile ND96 solution (96 mM NaCl, 2 mM KCl, 1 mM CaCl_2_, 1 mM MgCl_2_ and 5 mM HEPES at pH 7.4) supplemented with 5% FBS, 50 mg/L gentamycin (Sigma-Aldrich) and 100 μg/units/ml penicillin-streptomycin (Sigma-Aldrich). Electrophysiological recordings were carried out 2–6 days after microinjection.

### Dorsal root ganglion (DRG) neuron preparation

Rats were killed by cervical dislocation in accordance with the procedures approved by the Animal Ethics Committee of RMIT University. DRG neurons were enzymatically dissociated from ganglia of 4–21 day-old Wistar rats as described previously^14^. The spinal column was hemi-segmented and the paravertebral thoracic and lumbar ganglia were removed. Ganglia were rinsed in ice-cold Hanks’ balanced salt solution (HBSS, Life Technologies, Carlsbad, CA, USA) and incubated in 1.5 mg/ml collagenase (type 2; 340 U/mg) (Worthington Biochemical Corp., Lakewood, NJ, USA) in HBSS at 37 °C for 30 min. After incubation, ganglia were rinsed three times with pre-warmed (37 °C) Dulbecco’s Modified Eagle’s medium (DMEM, Life Technologies) supplemented with 10% fetal calf serum and 1% penicillin/streptomycin, and were triturated with a series of three fire-polished Pasteur pipettes of decreasing tip diameters. Cells were plated on poly-D-lysine/laminin-coated 12 mm round coverslips (BD Biosciences, Bedford, MA, USA), incubated at 37 °C in high relative humidity (95%) and controlled CO_2_ level (5%), and used within 4–48 h.

### Human embryonic kidney (HEK) cell culture and transfections

HEK-293 cells stably expressing human Ca_v_2.3c (R-type) channel α_1E-c_ splice variant (GenBank accession no. L29385), human α_2b_δ-1 (GenBank accession no. M76559) and human β_3a_ (RefSeq accession no. NM_000725) auxiliary subunits, were cultured as previously described^33^. The cells were transiently cotransfected with plasmid cDNAs encoding human γ-aminobutyric acid type B (GABA_B_) receptors, GABA_B_ R1 (RefSeq accession no. NM_001470; 3 μg; OriGene Technologies, Inc.) and GABA_B_R2 (RefSeq accession no. NM_005458; 3 μg; OriGene Technologies, Inc.), and enhanced green fluorescent protein (eGFP) reporter gene construct (1 μg), using the calcium phosphate precipitation method as described previously^33^. After transfection, cells were plated on glass coverslips incubated at 37 °C in high relative humidity (95%), and controlled CO_2_ level (5%). Transfection medium was then replaced with culture medium, and cells were incubated at 30 °C in in high relative humidity (95%) and 5% CO_2_.

### Electrophysiological recordings

Two-electrode voltage clamp recordings from oocytes were carried out at room temperature using a GeneClamp 500B amplifier (Molecular Devices Corp., Sunnyvale, CA) at a holding potential −80 mV. Voltage-recording and current-injecting electrodes were pulled from borosilicate glass (GC150T-7.5, Harvard Apparatus Ltd., Holliston MA) and had resistances of 0.3–1 MΩ when filled with 3 M KCl. A continuous push/pull syringe pump perfusion system was used to perfuse oocytes with ND96 at a rate of 2 ml/min. nAChR–mediated currents were evoked by application of 10 μM acetylcholine (ACh) at a rate of 2 ml/min via the perfusion system. Washout periods of 180–240 s between applications of ACh were used. Oocytes were incubated with peptides for 4 minutes before ACh was co-applied. Solutions of hcVc1.1 and the ACh control contained 0.1% bovine serum albumin. Peak ACh-evoked current amplitude was recorded before and after peptide incubation using pClamp 9 software (Molecular Devices Corp.).

Membrane currents in rat DRG neurons and HEK-293 cells were recorded using the whole-cell configuration of the patch clamp technique with an Axopatch 700B amplifier (Molecular Devices Corp., Sunnyvale, CA). For DRG neurons, the external recording solution contained the following (in mM): 150 tetraethylammonium (TEA)-Cl, 2 BaCl_2_, 10 D-glucose and 10 HEPES, pH 7.3. Fire-polished recording electrodes were filled with an internal solution containing (in mM): 140 CsCl, 1 MgCl_2_, 4 MgATP, 0.1 Na-GTP, 5 1,2-bis(O-aminophenoxy)ethane-N,N,N′,N′-tetraacetic acid tetracesium salt (BAPTA)-Cs_4_, and 10 HEPES-CsOH, pH 7.3, and had resistances of 1.5–2.2 MΩ. During recording, DRG neurons were constantly perfused with external recording using a gravity-fed perfusion system at a flow rate of ~1 ml/min.

HEK 293 cells were superfused with a solution containing (mM): 110 NaCl, 10 BaCl_2_, 1 MgCl_2_, 5 CsCl, 30 TEA-Cl, 10 D-glucose, and 10 HEPES, pH 7.4 with TEA-OH, at ~1 ml/min. Fire-polished recording electrodes with tip resistance values of 2–3 MΩ were filled with an intracellular solution containing (mM): 125 K-gluconate, 2 MgCl_2_, 5 EGTA, 5 NaCl, 4 MgATP, and 10 HEPES, pH 7.25 with CsOH. Depolarization-activated Ba^2+^ currents (I_Ba_) were elicited by 0.1 Hz, 120-ms step depolarizations to 0 mV, from a holding potential of −80 mV. Currents were filtered at 3 kHz and sampled at 10 kHz using pClamp 9.2 software in combination with Digidata 1322A (Molecular Devices). Leak and capacitive currents were subtracted using a −P/4 pulse protocol. Solutions with hcVc1.1 and baclofen were prepared from stock solutions and applied via perfusion in the bath solution.

### Curve fitting and statistical analysis

The concentration-response curves of nAChR–mediated currents in oocytes were obtained by plotting averaged relative peak current amplitude values (I/I_control_) against peptide concentration. The data was fitted by the Hill equation I = I_control_{[CTX]^n^/(IC_50_^n^ + [CTX]^n^)}, where I_control_ is the maximum peak current amplitude, [CTX] the conotoxin concentration, *n* the Hill coefficient, and IC_50_ the peptide concentration that inhibits 50 % of the maximum response (*n* = 3 to 6 for each data point).

In DRG neurons and HEK cells, the baclofen-sensitive fraction of I_Ba_ (I_baclofen-sensitive fraction_) was defined using the total I_Ba_ (peak I_Ba_ in the absence of a compound, I_control_) and the baclofen-resistant fraction (peak I_Ba_ in the presence of 50 μM baclofen, I_baclofen resistant_), as follows, I_baclofen-sensitive fraction_ = 1 − I_baclofen-resistant_/I_control_. Relative peak I_Ba_ amplitude values, I, were obtained by normalizing peak I_Ba_ values in the presence of hcVc1.1 (I_hcVc1.1_) to the I_baclofen-sensitive fraction_, as follows, I = I_hcVc1.1_ – I_baclofen resistant_/I_control_ − I_baclofen resistant_. Concentration–response curves were obtained by plotting I values versus hcVc1.1 concentration and fitting the above Hill equation to resulting data. Data are mean ± SEM (*n*, number of experiments).

## Additional Information

**How to cite this article**: Yu, R. *et al*. Less is More: Design of a Highly Stable Disulfide-Deleted Mutant of Analgesic Cyclic a-Conotoxin Vc1.1. *Sci. Rep*. **5**, 13264; doi: 10.1038/srep13264 (2015).

## Supplementary Material

Supplementary Information

## Figures and Tables

**Table 1:** IC_50_ values of synthetic α-conotoxins Vc1.1, cVc1.1 and hcVc1.1 for inhibition of rat DRG neuron N-type (Ca_v_2.2) channels, human Ca_v_2.3 and human α9α10 nAChRs.

Peptide	IC_50_ (nM)		
	rN-type (Ca_v_2.2)	hCa_v_2.3	hα9α10 nAChR
Vc1.1	1.7^a^	ND	320^d^
cVc1.1	0.3^c^	0.29^b^	6,000^d^
hcVc1.1	0.86^c^	0.96^d^	13,000^d^

Table shows mean values. ND, not determined. Superscript letters refer to references as follows.

^a^Callaghan *et al*., 2008^14^.

^b^Berecki *et al*., 2014^33^.

^c^Clark *et al*., 2010^9^.

^d^This study.
