# Supplementary material for: Less is More: Design of a Highly Stable Disulfide-Deleted Mutant of Analgesic Cyclic α-Conotoxin Vc1.1
Source: Sci Rep. 2015 Aug 20;5:13264. doi: 10.1038/srep13264 (PMC4542547; doi:10.1038/srep13264)
Supplement: Supplementary Information [file srep13264-s1.pdf]

Supplementary Information

Less is More: Design of a Highly Stable Disulfide-Deleted Mutant of Analgesic Cyclic α-Conotoxin Vc1.1

Rilei Yu, Victoria A.L. Seymour, Géza Berecki, Xinying Jia, Muharrem Akcan, David J. Adams, Quentin Kaas, and David J. Craik

Table S1. Molecular weight (MW) of oxidized hcVc1.1 and linear and cysteine ACM protected hcVc1.1 (“linear hcVc1.1”).

Peptide	Observed MW		Calculated MW	
	(*m*/*z* $[M+2H]^{2+}$)	(*m*/*z* $[M+2H]^{3+}$)	(*m*/*z* $[M+2H]^{2+}$)	(*m*/*z* $[M+2H]^{3+}$)
Linear hcVc1.1	1203.8	802.7	1203.5	802.66
Oxidized hcVc1.1	1120.7	747.3	1121.19	747.8

Table S2. Energy and structural statistics for the family of 20 models representing the solution structures of hcVc1.1. The values of the energies and RMSD are calculated using CNS.

**Energies (kcal/mol):**	
Overall	-895.02 ± 13.91
Bond	3.10 ± 0.38
Angle	18.19 ± 4.60
Improper	3.46 ± 1.08
Van der Waals	-39.89 ± 5.03
NOE	0.58 ± 0.50
cDih	0.03 ± 0.08
Dihedral	55.78 ± 8.16
Electrostatic	-936.27 ± 16.91
**RMSD:**	
Bond (Å)	0.0030 ± 0.002
Angles (degrees)	0.47 ± 0.06
Improper (degrees)	0.36 ± 0.06
NOE	0.007 ± 0.003
cDih	0.05 ± 0.07
**Pairwise RMSD:**	
Backbone/heavy, residues 1-16 (Å)	0.32±0.097
**Experimental data:**	
Distance restraints	135
Dihedral restraints	22
NOE violations exceeding 0.2 Å	0
cDih violations exceeding 2 (degree)	0
**Ramachandran (Molprobity):**	
Favoured regions	86.50%
Allowed regions	98.00%

Table S3. Amide proton temperature coefficients ΔδHN/ΔT (ppb/K) for hcVc1.1, cVc1.1 and Vc1.1 in 10% D2O 90% H2O (measured at 280–310 K).

Residue	hcVc1.1	cVc1.1[a]	Vc1.1[a]
G1	-7.13	-6.5	
H2/C2	-8.52	-5.1	-4.7
C3	-1.27	-5.6	-6.4
S4	-0.76	-2.3	-2.1
D5	-2.29	-3.5	-4.2
R7	-1.24	-2.1	-2.9
F8/C8	-2.6	-3.6	-4.3
N9	-6.14	-6.4	-7.6
Y10	-3.02	-4.5	-5.3
D11	-4.22	-3.9	-4.7
H12	0.37	-2.2	-2.6
E14	-2.32	-3.8	-4.6
I15	-1.37	-2.2	-2.6
C16	-4.95	-2.9	-3.5
G17	-4.02	-4.3	
G18	-6.05	-6.3	
A19	-4.72	-5.1	
A20	-7.8	-4.5	
G21	-8	-4.8	

[a] Values are taken from : Clark RJ, *et al.* (2010) The engineering of an orally active conotoxin for the treatment of neuropathic pain. *Angew Chem Int Ed Engl* 49(37):6545–6548.

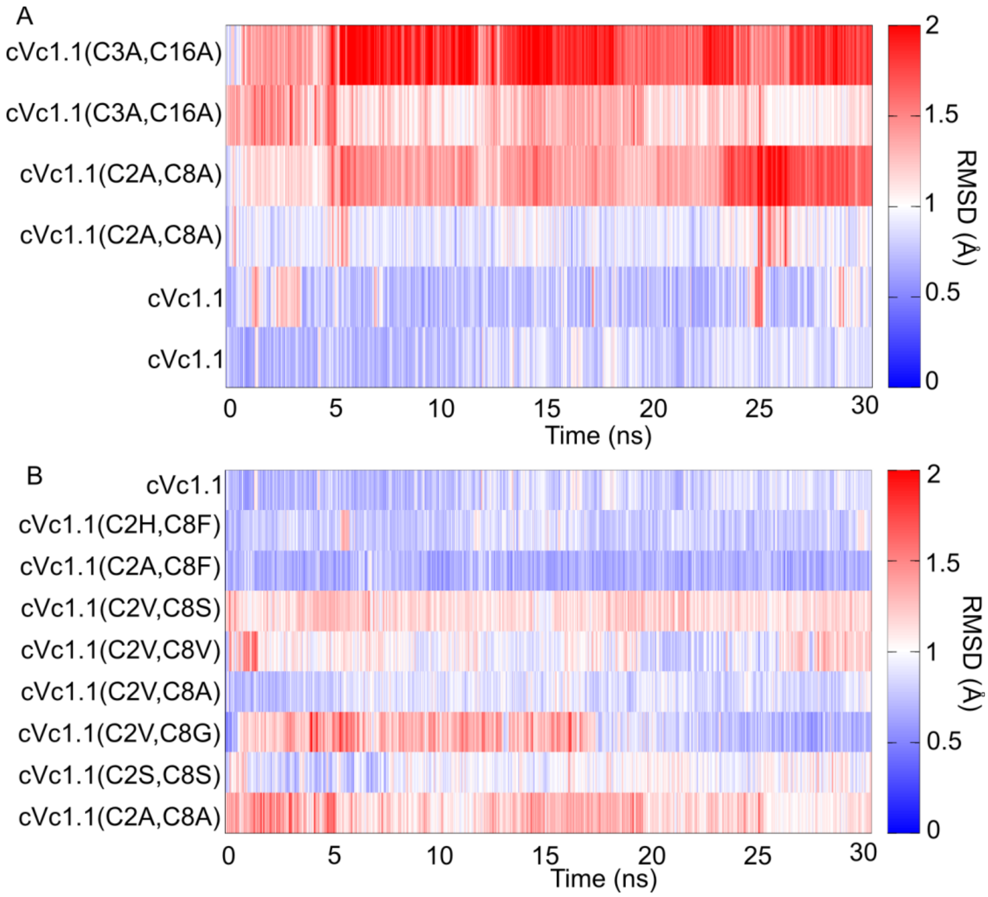

Figure S1. Evolution of the Cα RMSD of cVc1.1 and its variants from the NMR structure of cVc1.1 over 30 ns of molecular dynamics simulations. The RMSD was calculated using the Cα atom of residues 1–16. A: First round of design in which one cystine residue was replaced by alanine residues. B: Second round of design in which cystine 2–8 was replaced by various types of amino acids.

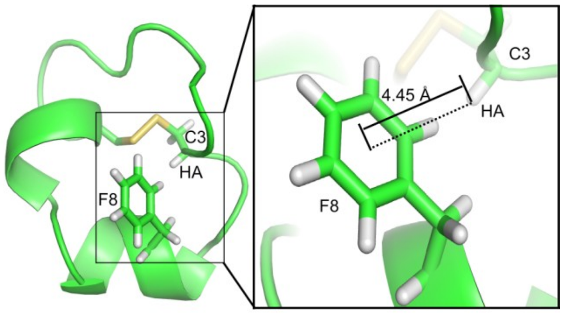

Figure S2. The ring current effect on the Hα chemical shift of the Cys-3. Panel A shows the relative orientation of Cys-3 and Phe-8. The Hα of Cys-3 is in the same plane as the aromatic ring of the Phe-8. The distance between Cys-3 Hα and the enter of the aromatic ring of Phe-8 is of 4.45 Å.

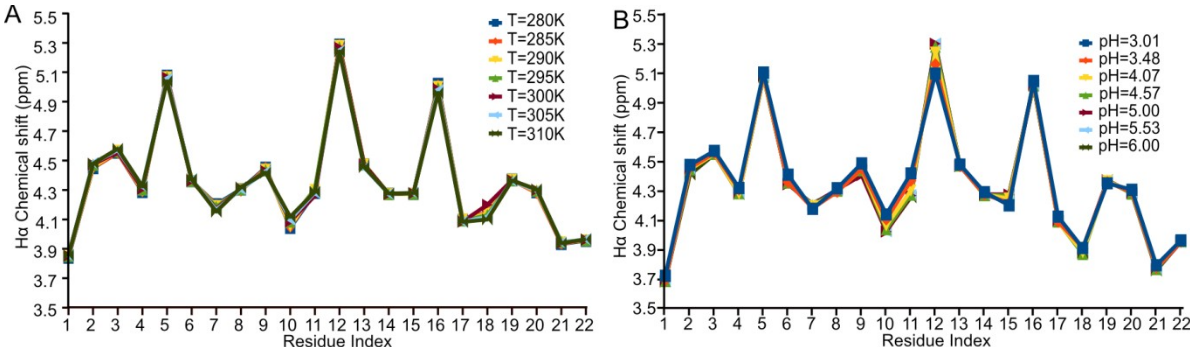

Figure S3. Influence of temperature (A) and pH (B) on hcVc1.1 Hα chemical shifts.

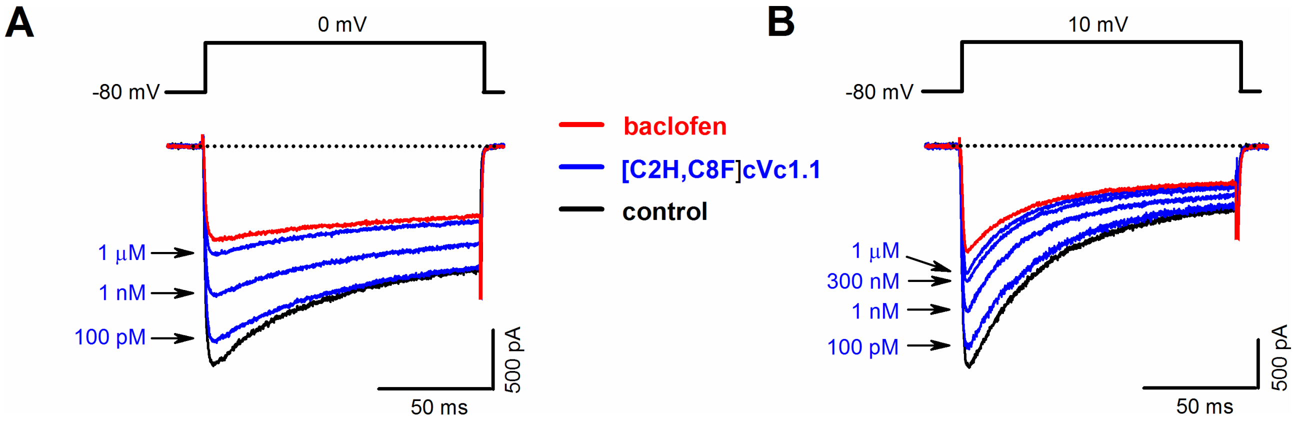

Figure S4. Representative superimposed whole-cell Ba^{2+} current (I_{Ba}) traces from a DRG neuron (A) and a HEK cell co-expressing human $Ca_v2.3$ calcium channels and human $GABA_B$ receptor subunits (B). I_{Ba} were recorded in the absence (*control*) and presence of increasing concentrations of hcVc1.1 (*arrowheads*). Baclofen (50 µM) was applied to determine the baclofen-sensitive I_{Ba} fraction. I_{Ba} were elicited at 0.1 Hz by 120-ms step depolarizations to 0 mV from a holding potential of −80 mV (*top* inset). Dotted line indicates zero-current level.

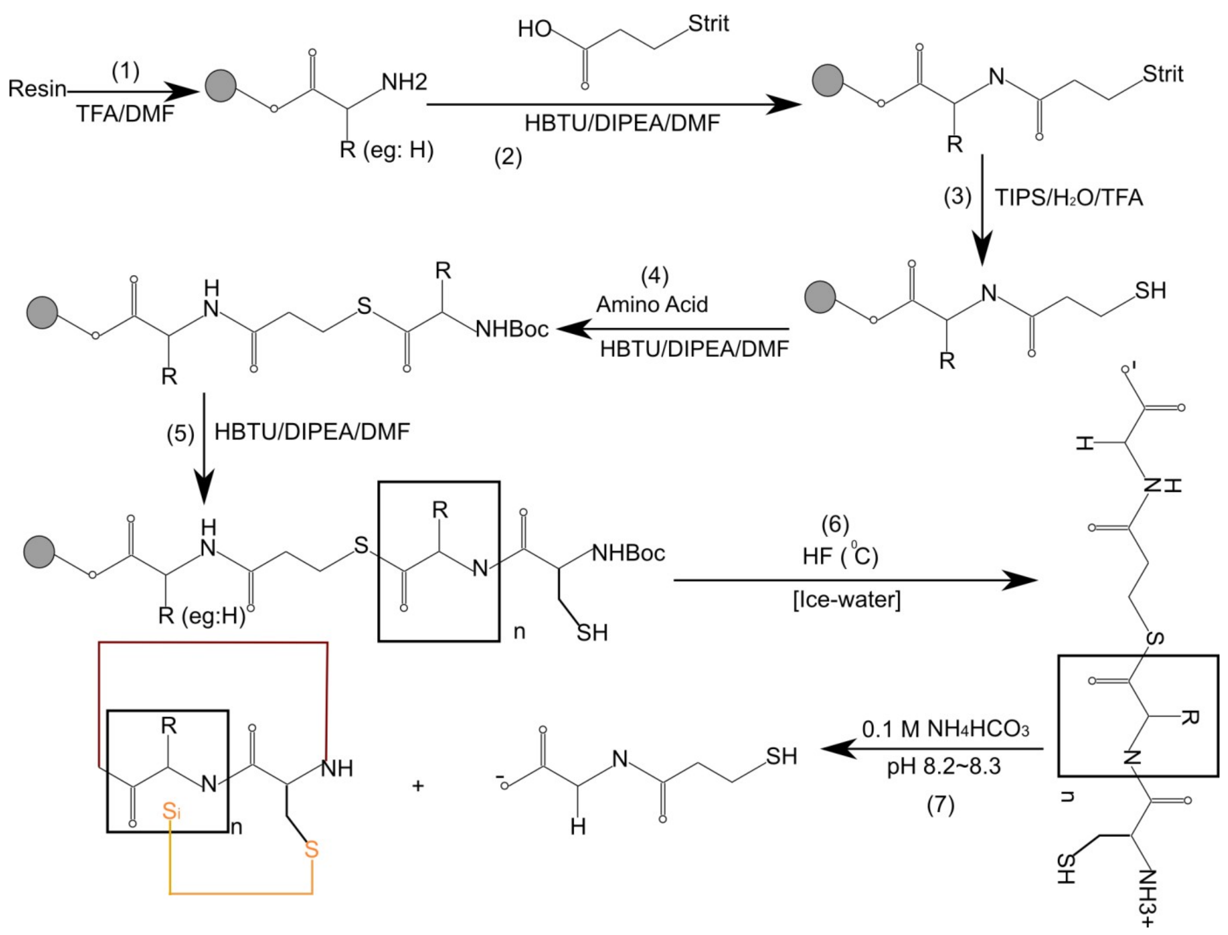

Figure S5. Chemical synthesis of hcVc1.1. The peptide was assembled on a Boc-Gly-Pam resin using Boc chemistry. (1) cleavage of protecting group of the Pam resin using TFA; (2), coupling of S-Tri linker to resin; (3), deprotection of linker; (4), coupling of the first amino acid to linker; (5), repeat step (3) and (4) until all residues are assembled; (6), cleavage of the linear peptide from resin using HF; (7), peptide oxidation and cyclization in NH_4HCO_3 solvent.

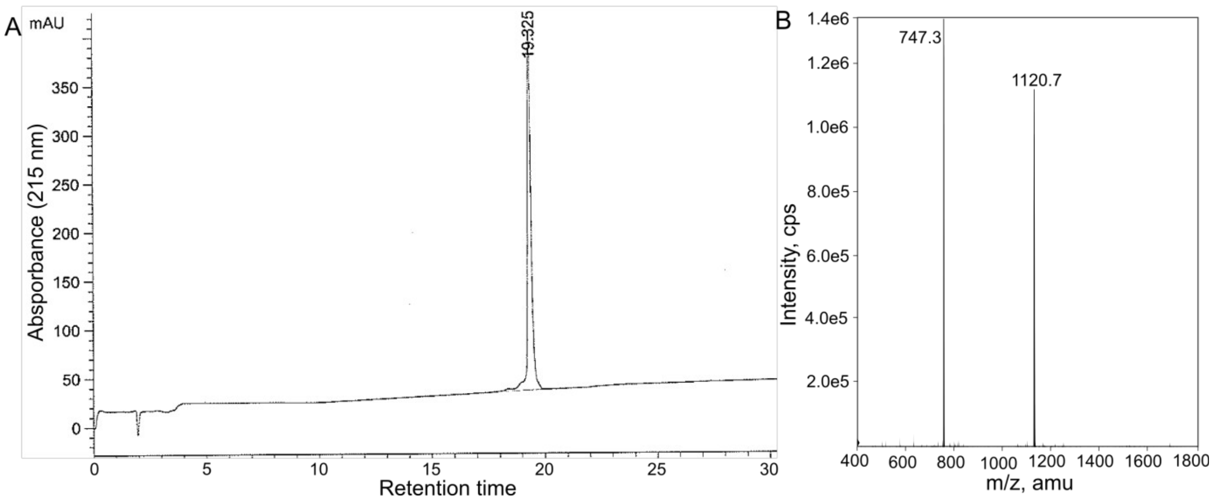

Figure S6. Characterization of oxidized hcVc1.1 using analytical HPLC and ES-MS. A: Purity of the peptide analyzed using analytical HPLC. B: Molecular weight validated using ES-MS.
